# Expression of HIV-1 Intron-Containing RNA in Microglia Induces Inflammatory Responses

**DOI:** 10.1128/JVI.01386-20

**Published:** 2021-02-10

**Authors:** Hisashi Akiyama, Sallieu Jalloh, Seonmi Park, Maohua Lei, Gustavo Mostoslavsky, Suryaram Gummuluru

**Affiliations:** a Department of Microbiology, Boston University School of Medicine, Boston Massachusetts, USA; b Center for Regenerative Medicine (CReM), Section of Gastroenterology, Department of Medicine, Boston University School of Medicine, Boston Massachusetts, USA; Icahn School of Medicine at Mount Sinai

**Keywords:** HIV, iPSC, intron-containing RNA, microglia, neuroinflammation

## Abstract

Chronic neuroinflammation is observed in HIV^+^ individuals on suppressive combination antiretroviral therapy (cART) and is thought to cause HIV-associated neurocognitive disorders. We recently reported that expression of HIV intron-containing RNA (icRNA) in productively infected monocyte-derived macrophages induces proinflammatory responses. Microglia, yolk sac-derived brain-resident tissue macrophages, are the primary HIV-1-infected cell type in the central nervous system (CNS). In this study, we tested the hypothesis that persistent expression of HIV icRNA in primary human microglia induces innate immune activation. We established multiple orthogonal primary human microglia-like cell cultures, including peripheral blood monocyte-derived microglia (MDMG) and induced pluripotent stem cell (iPSC)-derived microglia. Unlike MDMG, human iPSC-derived microglia (hiMG), which phenotypically mimic primary CNS microglia, were robustly infected with replication-competent HIV-1, and establishment of productive HIV-1 infection and *de novo* viral gene expression led to proinflammatory cytokine production. Blocking of HIV-1 icRNA expression, but not multiply spliced viral RNA, via either infection with virus expressing a Rev-mutant deficient for HIV icRNA nuclear export or infection in the presence of a small molecule inhibitor of the chromosome region maintenance 1 (CRM1)-mediated viral icRNA nuclear export pathway attenuated induction of innate immune responses. These studies suggest that Rev-CRM1-dependent nuclear export and cytosolic sensing of HIV-1 icRNA induces proinflammatory responses in productively infected microglia. Novel strategies targeting HIV icRNA expression specifically are needed to suppress HIV-induced neuroinflammation.

**IMPORTANCE** Although peripheral viremia can be effectively suppressed with the advent of highly active antiretroviral therapy, a significant portion of HIV^+^ individuals still suffer from neurocognitive disorders. Despite suppressive therapy, HIV persists in various tissues, including the central nervous system (CNS), leading to chronic inflammation, the chief driver of neurocognitive disorders. While persistent infection has been described in CNS-resident macrophages and microglia, molecular mechanisms of how HIV infection in microglia contributes to neuronal inflammation have remained unclear. In this study, we used multiple primary human microglia-like cellular platforms and demonstrate that HIV-1 intron-containing RNA induces microglial activation and damage. Since current antiretroviral therapy does not suppress HIV-1 transcription, new therapeutics targeting HIV RNA expression may help to treat HIV-associated neurocognitive disorders.

## Abstract (precis)

Although peripheral viremia can be effectively suppressed with the advent of highly active antiretroviral therapy, a significant portion of HIV^+^ individuals still suffer from neurocognitive disorders. Despite suppressive therapy, HIV persists in various tissues, including the central nervous system (CNS), leading to chronic inflammation, the chief driver of neurocognitive disorders.

## INTRODUCTION

Since the advent of combination antiretroviral therapy (cART), mortality and morbidity of HIV-1 infection has been dramatically reduced. Although prolonged cART can suppress peripheral viremia in HIV^+^ individuals under the detection limit for decades, these therapeutic regimens fail to suppress chronic immune activation, the chief driver of HIV-associated non-AIDS complications (HANA), including HIV-associated neurocognitive disorders (HAND) (1, 2). Numerous studies have demonstrated that inflammatory markers associated with myeloid cell activation are strongly and selectively predictive of HAND (3). *In vivo*, persistent HIV infection has been reported in the central nervous system (CNS)-resident macrophages, including perivascular macrophages and microglia (4–7). However, molecular mechanisms of how HIV infection in the CNS-resident macrophages contributes to chronic immune activation have remained unclear.

Recently, we showed that expression and Rev-CRM1-dependent nuclear export of HIV intron-containing RNA (icRNA) in productively infected peripheral blood monocyte-derived macrophages (MDMs) is the trigger to induce type I interferon (IFN-I)-dependent production of proinflammatory cytokines even in the absence of new viral particle production (8). Similar findings have also been reported for monocyte-derived dendritic cells (9), suggesting HIV icRNA expression-induced innate immune activation might be a conserved phenotype in myeloid cells. Numerous studies have documented the continued presence of HIV RNA in the cerebrospinal fluid (CSF) even after prolonged cART (3, 10–12). Since cART regimens as constituted presently cannot suppress viral RNA expression from integrated proviruses, it is plausible that persistent expression of HIV icRNA in the CNS-resident microglia and perivascular macrophages contributes to the chronic inflammatory state in the brain of HIV^+^ individuals on cART.

Productively infected microglia can contribute to virus persistence and CNS pathology during HIV-1 infection (4, 13), though the extent to which these reservoirs persist and the mechanisms that might allow for virus persistence in these cells in patients on cART remain unclear. HIV infection of microglia has been shown to impact microglial functions, including activation status, viability, and metabolism (14). In addition, changes in microglial functions have been postulated to contribute to neuropathogenesis by secreting proinflammatory cytokines and neurotoxins (15). Activated microglia are also known to cause neurodegeneration directly by damaging synapses or indirectly via activation of other CNS-resident cells such as astrocytes (reviewed in reference 16). Microglia play a pivotal role in maintaining brain homeostasis, and microglial dysfunction caused by HIV infection is thought to impact CNS functionality of HIV^+^ individuals on suppressive cART. To date, several mechanisms have been proposed to explain how HIV induces microglia activation. For example, the HIV proteins Tat, gp120, Nef, and Vpr have been shown to activate microglia, leading to alterations in microglial functions and neuronal health (reviewed in reference 14). However, the physiological relevance of these findings needs to be carefully considered, since most of the studies used overexpression of viral proteins or transgenic rodents. Whether such high concentrations of these viral proteins are observed in the CNS of HIV^+^ patients on suppressive therapy requires further investigation. While HIV infection of primary human fetal microglia has been reported (17, 18), these cells are not easily accessible, which precludes detailed investigations of the molecular mechanisms of HIV-induced innate immune activation. Overall, the molecular mechanisms of HIV-induced microglia activation in the CNS remain unclear.

In this study, we investigate the role of HIV-1 infection of microglia in promoting neuroinflammation using two model systems, primary monocyte-derived microglia (MDMG) and induced pluripotent stem cell (iPSC)-derived microglia (iCell-MG and hiMG). We report that while HIV-1 infection of MDMGs is attenuated, restriction to infection was alleviated upon SAM domain and HD domain-containing protein 1 (SAMHD1) degradation. In contrast, both iCell-MGs and hiMGs were robustly infected with wild-type HIV-1, and innate immune activation in these cells was triggered by *de novo* expression and nuclear export of icRNA via the Rev-CRM1-dependent pathway.

## RESULTS

### MDMG model of HIV-1 infection in microglia.

**FIG 1:**
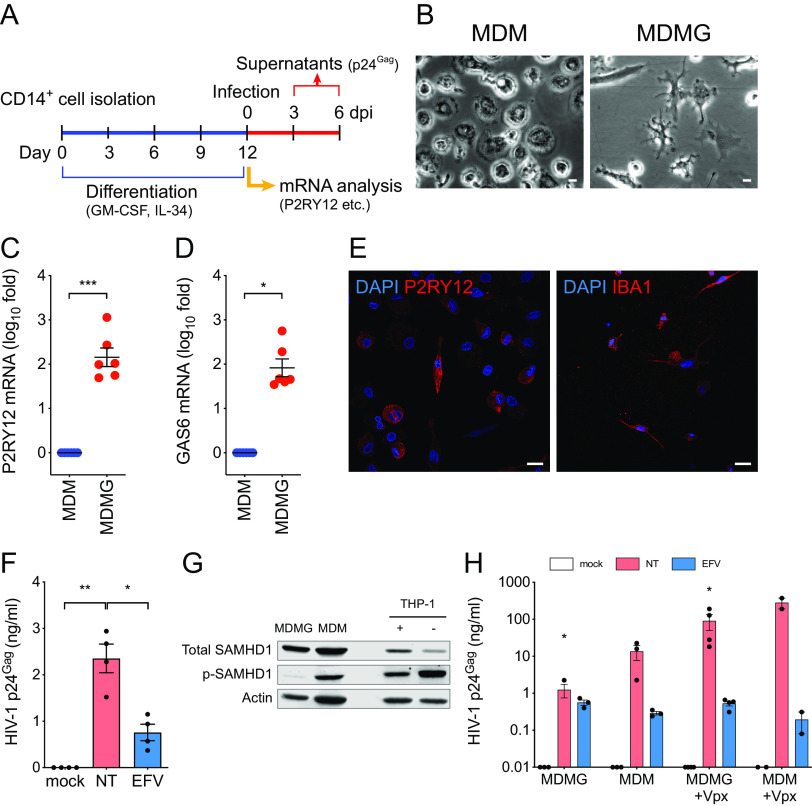
Monocyte-derived microglia (MDMG) are susceptible to HIV-1 infection. (A) Schematic of MDMG differentiation protocol. (B) Representative image of MDMs or MDMGs differentiated from the same donor. Bars = 20 µm. (C and D) Expression of (C) P2RY12 and (D) GAS6 mRNA in MDMGs was quantified by reverse transcription-quantitative PCR (qRT-PCR) and normalized to that of MDM generated from the same donor. (E) Representative immunofluorescence images of MDMGs stained for nucleus (DAPI, blue) and P2RY12 or IBA-1 (red). Bar = 20 µm. (F) MDMGs were infected with Lai/YU-2env (replication-competent CCR5-tropic HIV-1, MOI = 1), and production of p24^Gag^ in the culture supernatant was quantified by ELISA (3 days postinfection [dpi]). (G) Western blot analysis for total SAMHD1, phosphorylated SAMHD1 expression in MDMGs, MDMs and THP-1 cells. Actin was probed as a loading control. +, PMA-treated THP-1; –, unstimulated THP-1. (H) MDMGs and MDMs were infected with HIV-1 (LaiΔenvGFP/VSV-G, MOI = 2, in the absence or presence of SIV_mac_239 Vpx VLPs), and production of p24^Gag^ in the culture supernatant was quantified by ELISA (3 dpi). NT, no treatment (DMSO); EFV, efavirenz (1 µM); Ral, raltegravir (30 µM). The means ± standard error of the mean (SEM) are shown, and each symbol represents an independent experiment. *P* values: one-sample *t* test (panel C, two-tailed), the Wilcoxon matched-pairs signed rank test (panel D, two-tailed), or one-way ANOVA followed by the Tukey-Kramer posttest (panel F) or Dunnett’s posttest comparing to mock (panel H). *, *P* < 0.05; **, *P* < 0.01; ***, *P* < 0.001.

HIV-1 infection of primary human fetal microglia has been reported (17, 18), though these cells are not easily accessible due to ethical and technical issues. To overcome these limitations, microglia-like cells have been generated *in vitro* from monocytes and characterized extensively (19–22). We derived microglia-like cells from CD14^+^ monocytes by culturing in serum-free conditions in the presence of interleukin-34 (IL-34) and granulocyte-macrophage colony-stimulating factor (GM-CSF) (Fig. 1A). These cells displayed a unique microglia-like ramified morphology (Fig. 1B), as previously reported (19, 20). MDMGs have been shown to display similar morphology to that of human primary microglia and express genes that are highly or uniquely expressed in human microglia (19–23). In agreement with these previous findings, expression of P2RY12 and *Gas6* mRNAs in MDMGs was significantly enhanced compared to those in donor-matched monocyte-derived macrophages (MDMs) (Fig. 1C and D). Furthermore, expression of *P2RY12* and IBA-1 in MDMGs was confirmed by immunofluorescence (Fig. 1E). We next examined if MDMGs were susceptible to HIV-1 infection. MDMGs were infected with replication-competent-tropic HIV-1 (Lai/YU-2env), and p24^Gag^ secretion in the culture supernatants was quantified by enzyme-linked immunosorbent assay (ELISA). While infection of MDMGs resulted in productive infection and release of progeny virions (Fig. 1F), the amount of p24^Gag^ in the supernatants was low. Since MDMGs were differentiated from peripheral blood monocytes in GM-CSF- and IL-34-containing media, and GM-CSF has been shown to alter the phosphorylation status of SAMHD1 and render MDMs less susceptible to HIV-1 infection (24), we sought to determine the phosphorylation status of SAMHD1 in MDMGs. Western blotting demonstrated that while total SAMHD1 levels were similar, MDMGs expressed significantly reduced levels of phosphorylated SAMHD1 compared to donor-matched MDMs or THP-1 macrophages (Fig. 1G) (25, 26). We next infected MDMGs and donor-matched MDMs with HIV-1 in the absence or presence of the simian immunodeficiency virus of macaques (SIV_mac_) Vpx containing virus-like particles (VLPs), which degrades SAMHD1 (27, 28) and enhances HIV-1 infection of myeloid cells (29). In the absence of SIV_mac_ Vpx, MDMGs produced a much smaller amount of p24^Gag^ in the supernatants than MDMs (Fig. 1H). Interestingly, pretreatment of MDMGs with SIV_mac_ Vpx VLPs significantly enhanced p24^Gag^ production (Fig. 1H), suggesting that abundant expression of antiviral SAMHD1 in MDMGs restricts efficient infection of these cells by HIV-1.

### HIV-1 infection induces immune activation in MDMGs.

**FIG 2:**
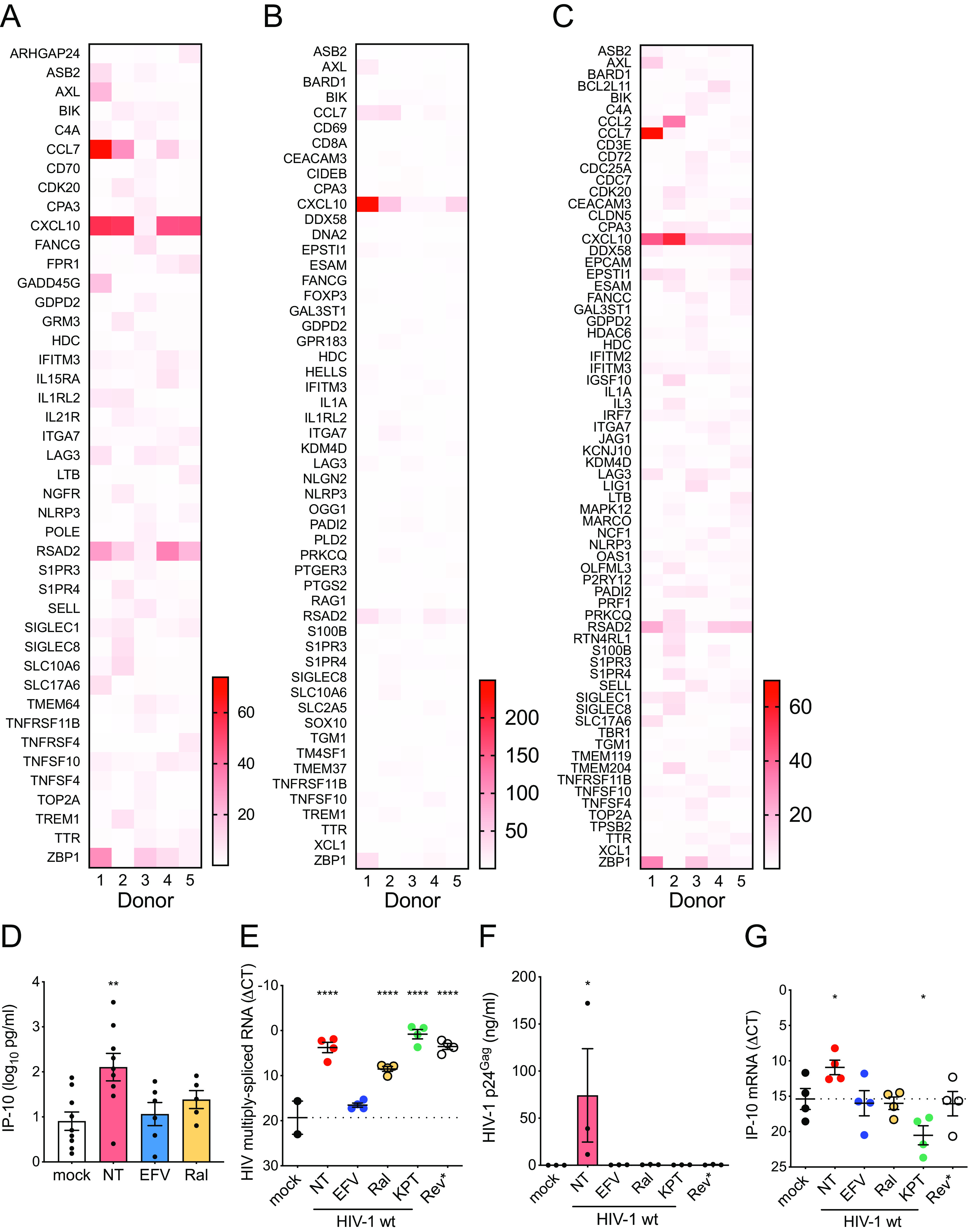
HIV-1 infection induces innate immune activation in MDMGs. (A) mRNA expression profiles in MDMGs infected with HIV-1 (LaiΔenvGFP/VSV-G, MOI = 2, in the presence of SIV_mac239_ Vpx VLPs) were analyzed using the human neuroinflammation panel (NanoString). (A to C) Expression of mRNA in HIV-1-infected MDMGs was normalized to that in mock-infected MDMGs (A), in infected MDMGs in the presence of (B) efavirenz or (C) raltegravir, and genes which were expressed more than the mean + 2 × standard deviation (SD) are shown. (D) Production of IP-10 in HIV-1-infected MDMGs (MOI = 2, 3 dpi) measured by ELISA. (E to G) Effects of CRM1 inhibitor (KPT-330) on HIV-1-infected MDMGs or infection of MDMGs with a Rev mutant deficient for icRNA nuclear export (Rev*, M10) on (E) viral infection (multiply spliced viral RNA expression, Rev-independent, shown as ΔCT to GAPDH), (F) p24^Gag^ production (Rev-dependent) measured by ELISA, or (G) IP-10 mRNA expression (shown as ΔCT to GAPDH). The means ± SEM are shown, and each symbol represents an independent experiment. *P* values: one-way ANOVA followed by Dunnett’s posttest comparing to mock (panels D to G). *, *P* < 0.05; **, *P* < 0.01; ****, *P* < 0.0001. NT, no treatment (DMSO); EFV, efavirenz (1 µM); Ral, raltegravir (30 µM); KPT, KPT-330 (Selinexor, 1 µM); Rev*, M10.

We recently showed that infection of MDMs with HIV-1 induces interferon I (IFN-I)-dependent proinflammatory responses (8). To investigate whether HIV-1 infection of microglia induces innate immune activation, total RNA isolated from HIV-1-infected MDMGs in the presence of SIV_mac_ Vpx VLPs was analyzed with a NanoString human neuroinflammation panel that contains more than 750 target genes covering the core pathways and processes involved in neuroinflammation. Among those analyzed, several mRNAs were upregulated in an HIV-1 infection-specific manner; i.e., upregulation was only seen in HIV-infected untreated MDMGs but not in reverse transcriptase inhibitor (efavirenz, EFV)- or integrase inhibitor (raltegravir, Ral)-treated MDMGs (Fig. 2A and B and C). Highly upregulated genes (>mean + 2 × SD) compared to mock-, EFV- or Ral-treated MDMGs are shown in Fig. 2A, B, and C, respectively, which include interferon-stimulated genes (ISGs) (e.g., Siglec1/CD169, RSAD2) and proinflammatory cytokines (e.g., CXCL10/IP-10, CCL7/MCP-3). To confirm the results from NanoString analysis, IP-10 production in the MDMG culture supernatants was measured by ELISA. We found that IP-10 production was induced upon infection of MDMGs with HIV-1, which was inhibited upon pretreatment of MDMGs with EFV or Ral (Fig. 2D). HIV-1 intron-containing RNA (icRNA) export into cytosol via the Rev-CRM1-dependent pathway has previously been shown to induce innate immune activation in MDMs and dendritic cells (8, 9). To investigate the role of HIV-1 icRNA export by the Rev-CRM1-dependent pathway in MDMG innate activation, HIV-1 infected MDMGs were treated with a CRM1 inhibitor (KPT-330, selinexor), or MDMGs were infected by an HIV-1 Rev-deficient (dominant negative) mutant (M10) (8, 30). While establishment of infection of MDMGs and HIV-1 multiply spliced RNA expression was not affected by KPT treatment or M10 infection (Fig. 2E), production of p24^Gag^ which is transcribed from icRNA, was completely inhibited by KPT-330 treatment or in M10-infected MDMGs (Fig. 2F). Interestingly, expression of IP-10 mRNA was severely reduced in HIV-1-infected MDMGs upon KPT-330 treatment or in M10-infected MDMGs (Fig. 2G). These results suggest that innate immune activation of MDMGs upon HIV-1 infection requires cytoplasmic expression of HIV icRNA exported via the Rev-CRM1-dependent pathway.

### iPSC-derived microglia are highly susceptible to HIV-1 infection.

**FIG 3:**
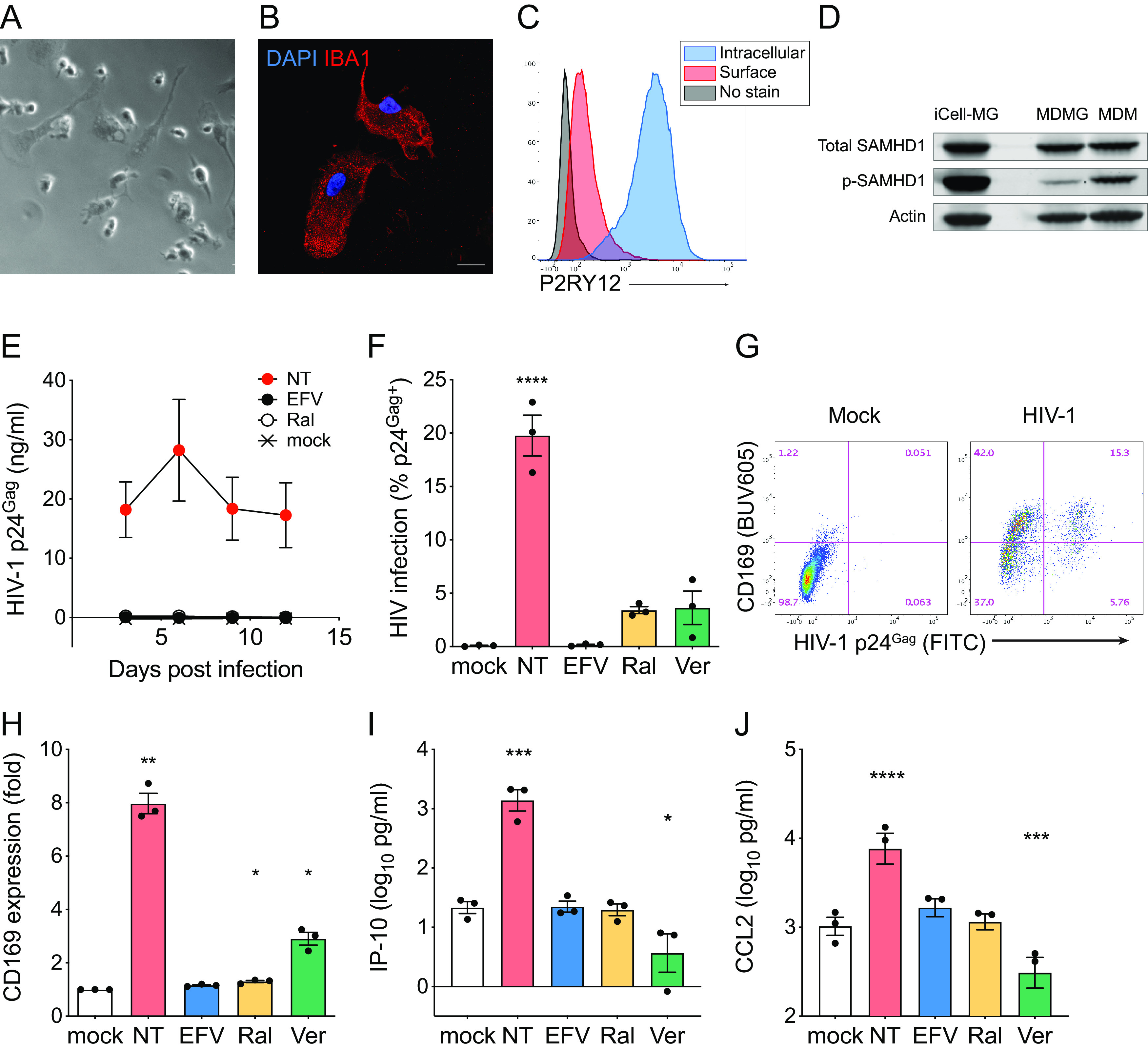
iPSC-derived microglia are highly susceptible to HIV-1 infection. (A) Representative phase-contrast images of iCell-MGs (Fujifilm Cellular Dynamics). Bar = 20 µm. (B) Representative immunofluorescence image of iCell-MGs stained for nucleus (DAPI, blue) and IBA-1 (red). Bar = 20 µm. (C) Representative flow cytometry profile of iCell-MGs stained for intracellular and surface P2RY12. (D) Western blot analysis for total SAMHD1, phosphorylated SAMHD1 expression in iCell-MGs, MDMGs, and MDMs. Actin was probed as a loading control. (E) Replication kinetics of HIV-1 in iCell-MGs. Cells were infected with HIV-1 (Lai/YU-2env, replication competent CCR5 tropic HIV-1, MOI = 1), and production of p24^Gag^ in the culture supernatant was quantified by ELISA. (F to H) iCell-MGs were infected with HIV-1 (Lai/YU-2env, MOI = 1), and (F) HIV-1 infection (intracellular p24^Gag^ expression) and (G and H) CD169 expression were analyzed by flow cytometry. (I and J) Production of the proinflammatory cytokines (I) IP-10 and (J) CCL2 in the culture supernatants was measured by ELISA (6 dpi). The means ± SEM are shown, and each symbol represents an independent experiment. *P* values: one-way ANOVA followed by Dunnett’s posttest comparing to mock (panels F and H to J), *, *P* < 0.05; **, *P* < 0.01; ***, *P* < 0.001; ****, *P* < 0.0001. NT,: no treatment (DMSO); EFV, efavirenz (1 µM); Ral, raltegravir (30 µM); Ver, verdinexor (KPT-335, 0.1 µM).

Fate mapping analysis suggests that microglia in the brain originate from yolk-sac-derived primitive macrophages during embryonic hematopoiesis (31, 32). Unlike other tissue-resident macrophages, such as Kupffer cells and alveolar macrophages, microglia are not replenished with circulating bone marrow-derived monocytes during adulthood (33–35). To better model HIV-1 infection of human primary microglia, we tested if human induced pluripotent stem cell (iPSC)-derived microglia can be infected with HIV-1. We obtained iPSC-derived microglia, iCell microglia (iCell-MG), from a commercial source (Fujifilm Cellular Dynamics), which were generated as previously described (36). iCell-MGs showed heterogeneous morphology (Fig. 3A) and expressed the macrophage/microglia marker IBA-1 (Fig. 3B). Flow cytometry analysis revealed robust intracellular expression of the microglia-specific marker P2RY12 and minimal expression on the cell surface (Fig. 3C). Immunoblotting analysis revealed that, in contrast to MDMGs, the majority of SAMHD1 was phosphorylated in iCell-MGs (Fig. 3D). We then infected iCell-MGs with replication-competent CCR5-tropic HIV-1/YU-2 and monitored p24^Gag^ production in the culture supernatants over 15 days. We found that iCell-MGs persistently produced p24^Gag^, which peaked at 6 days postinfection (p.i.) (Fig. 3E). Intracellular p24^Gag^ staining revealed that about 20% of iCell-MGs in the culture were productively infected at 6 days p.i. (Fig. 3F). HIV-1 replication in the infected iCell-MG cultures was inhibited by reverse transcriptase (efavirenz, EFV), integrase (raltegravir, Ral), and CRM1 (KPT-335, verdinexor) inhibitors (Fig. 3E and F). To investigate if HIV-1 infection of iCell-MGs induced innate immune activation, we harvested cells on day 6 p.i. and stained them for CD169, a myeloid-cell-specific ISG (37, 38). iCell-MGs upregulated CD169 expression upon infection with HIV-1 (Fig. 3G) on both infected cells and on bystander uninfected cells, suggesting that low levels of IFN-I were secreted by infected cells, similar to what was observed in HIV-1-infected MDMs (8). Expression of CD169 was suppressed by pretreatment of iCell-MGs with RT (EFV), integrase (Ral), and CRM1 (verdinexor) inhibitors (Fig. 3H). Furthermore, IP-10 and CCL2 production was induced by productive infection of iCell-MGs by HIV-1 and inhibited upon treatment by EFV, Ral, or verdinexor (Fig. 3I and J). These results suggest that iPSC-derived microglia are highly susceptible to HIV-1 infection and that expression and nuclear export of HIV icRNA in infected iCell-MGs triggers innate immune responses in microglia.

### Establishment of iPSC-derived microglia/neuron coculture system.

**FIG 4:**
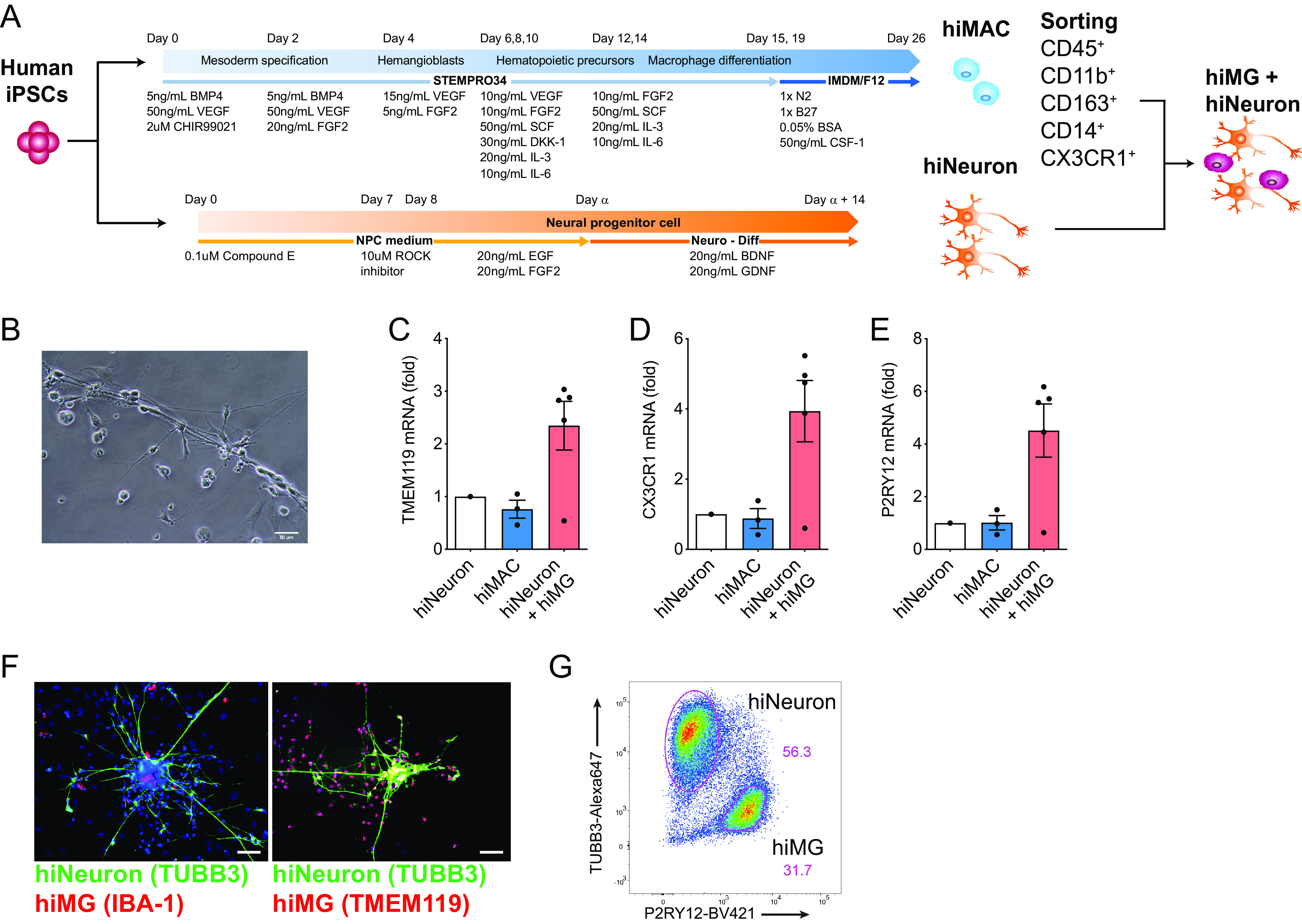
Establishment of iPSC-derived microglia/neuron coculture system. (A) Schematic of hiMG generation by coculturing hiMAC (yolk-sac-derived primitive macrophages) and neurons from human iPSCs. (B) Representative phase-contrast image of hiMGs and hiNeurons cocultured for 11 days. Bar = 50 µm. (C to E) Expression of (C) TMEM119, (D) CX3CR1, and (E) P2RY12 mRNA in hiMACs or hiMG-hiNeuron cocultures was quantified by qRT-PCR and normalized to that of hiNeuron solo culture. *P* values from one-way ANOVA test for panels C, D, and E were 0.0972, 0.0829, and 0.0814, respectively. (F) Representative immunofluorescence images of hiMG-hiNeuron cocultures stained for nucleus (DAPI, blue), neuron (tubulin beta 3: TUBB3, green), and MG markers IBA-1 or TMEM119 (red). Bars = 50 µm. (G) Representative flow cytometry profile of hiMG-hiNeuron coculture stained for neurons (TUBB3) and hiMGs (P2RY12). The means ± SEM are shown, and each symbol represents an independent experiment.

We took advantage of recent descriptions in the literature of generation of microglia from iPSC lines (39). Briefly, iPSC-derived human microglia were derived by coculturing iPSC-derived yolk-sac primitive macrophages (hiMAC) with iPSC-derived neurons (hiNeuron) (Fig. 4A and B) (39). Cells in the hiMG-hiNeuron cocultures expressed significantly higher levels of mRNA of the microglia-specific markers TMEM119 (Fig. 4C), CX3CR1 (Fig. 4D), and P2RY12 (Fig. 4E) compared to those in hiNeuron monococulture or in hiMACs. Immunofluorescence revealed that hiMGs expressed macrophage/microglia markers (IBA-1 or TMEM119) (39, 40) and made numerous cell-to-cell contacts with neurons as previously reported (Fig. 4F) (39). P2RY12 was highly expressed on the cell surface of hiMGs, similar to CNS-resident human microglia (23, 41), and these cells were clearly distinguishable from hiNeuron (tubulin β3/TUBB3^+^) by flow cytometry (Fig. 4G).

### HIV-1 infection of hiMGs induces proinflammatory responses.

**FIG 5:**
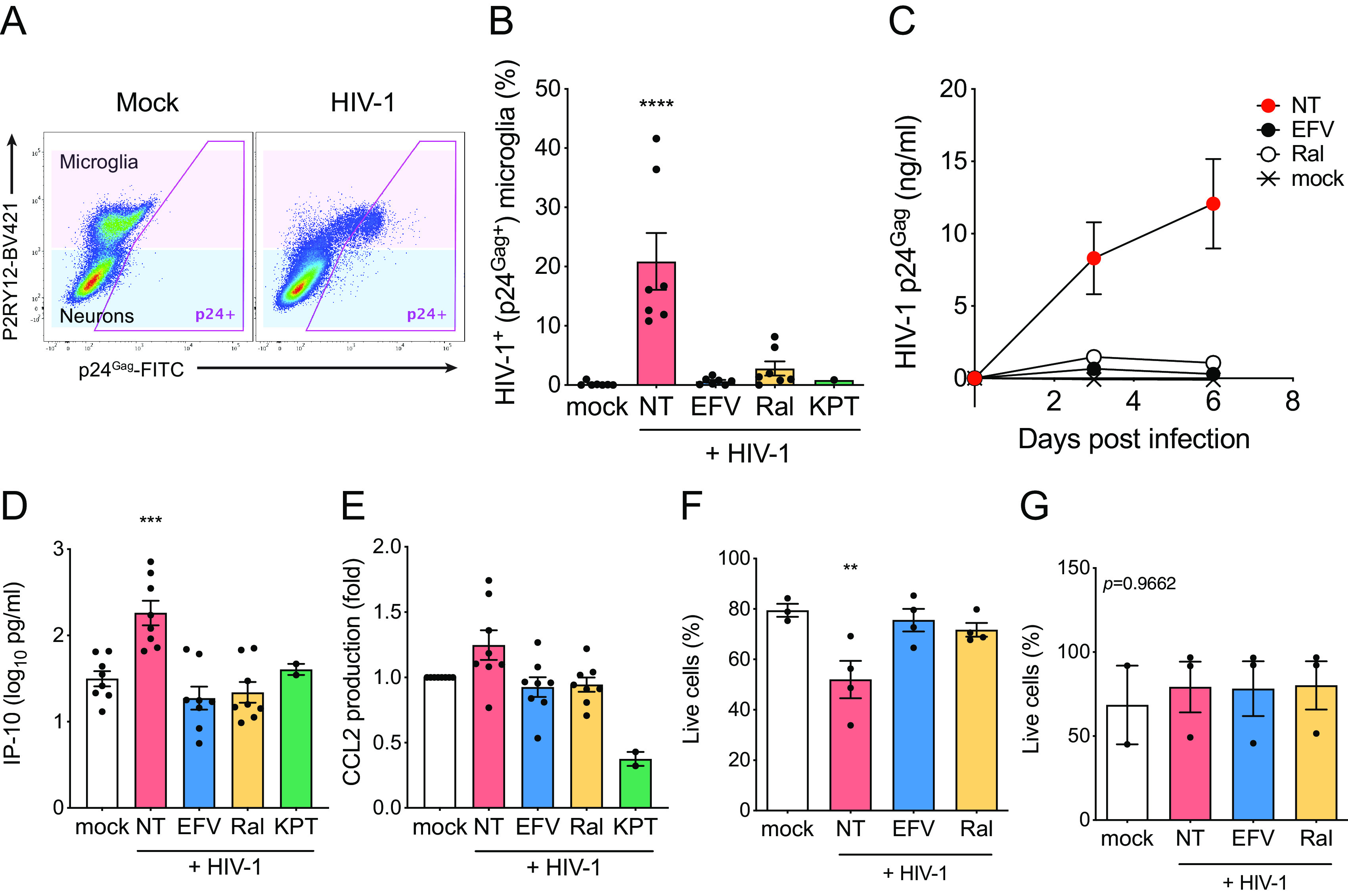
HIV-1 infection of hiMGs in hiMG-hiNeuron cocultures induces proinflammatory responses. hiMG-hiNeuron cocultures were infected with HIV-1 (Lai/YU-2env: replication-competent CCR5 tropic HIV-1, MOI = 1). (A and B) HIV-1 infection (intracellular p24^Gag^ expression) was analyzed by flow cytometry. (A) Representative flow cytometry profile is shown, and microglia (P2RY12^+^) and neuron (P2RY12^−^) populations are highlighted with pink and blue, respectively. (B) HIV-infected (p24^Gag+^) cells in microglia (pink in panel A) were calculated. (C) Replication kinetics of HIV-1 in hiMG-hiNeuron coculture. Cocultures were infected with HIV-1 (Lai/YU-2env, replication-competent CCR5 tropic HIV-1, MOI = 1), and production of p24^Gag^ in the culture supernatant was quantified by ELISA. (D and E) Production of proinflammatory cytokines (D) IP-10 and (E) CCL2 was measured by ELISA (6 dpi). (F and G) The proportion of live cells in (F) microglia (pink in panel A) and (G) neurons (blue in panel A) was calculated. The means ± SEM are shown, and each symbol represents an independent experiment. *P* values: one-way ANOVA followed by Dunnett’s posttest comparing to mock (panels B and D to F); **, *P* < 0.01; ***, *P* < 0.001; ****, *P* < 0.0001. The *P* value from one-way ANOVA test was 0.9662 for panel G. NT, no-treatment (DMSO); EFV, efavirenz (1 µM); Ral, raltegravir (30 µM); KPT, KPT-330 (selinexor, 1 µM).

The iMG-hiNeuron cocultures were infected with replication-competent HIV-1 Lai/YU-2env, and HIV-1 replication was measured by flow cytometry (intracellular p24^Gag^ expression) or ELISA (p24^Gag^ in the culture supernatants). While hiNeurons were not susceptible to HIV-1, hiMGs were robustly infected with HIV-1 in hiMG–hiNeuron cocultures (Fig. 5A and B). Furthermore, establishment of infection in hiMG-hiNeuron cocultures was blocked by pretreatment with EFV and Ral and anti-CRM1 inhibitor (KPT-330) (Fig. 5B). We detected increasing amounts of p24^Gag^ in the culture supernatants over time (Fig. 5C), which is suggestive of persistent virus replication in hiMG-hiNeuron cocultures. HIV-1 infection induced increased production of IP-10 (Fig. 5D) and upregulated CCL2 secretion (Fig. 5E). HIV-1 infection in microglia has been postulated to lead to neuronal disorder by disrupting microglia viability and functionality (14). To investigate the impact of HIV-1 infection on microglial functionality and neuronal toxicity, HIV-1-infected hiMG-hiNeuron cocultures were analyzed for microglial and neuronal viability by flow cytometry on day 6 p.i. Interestingly, the proportion of live microglia in the cocultures decreased upon HIV-1 infection over time, which was suppressed upon initiation of infections in the presence of HIV-1 inhibitors (EFV and Ral), suggesting that productive HIV-1 infection, but not exposure to HIV-1 particles alone, affected hiMG viability (Fig. 5F). On the other hand, HIV-1 spread in hiMG-hiNeuron cocultures did not affect the viability of hiNeurons (Fig. 5G). These data suggest that hiMGs in the microglia-neuron cocultures are highly susceptible to HIV-1 infection and that Rev-CRM1-dependent nuclear export of HIV icRNA in microglia triggers secretion of proinflammatory cytokines, which might contribute to neuroinflammation *in vivo*.

## DISCUSSION

### HIV infection and innate immune responses.

Chronic inflammation is thought to be the chief driver of HAND (2, 42, 43), though underlying mechanisms of persistent neuroinflammation remain unclear. In this study, we demonstrated that HIV-1 infection of microglia induces innate immune activation, resulting in secretion of proinflammatory cytokines, upregulation of ISGs, and microglia cytotoxicity. Considering their long life span with self-renewal capacity (31, 44, 45), coupled with the observation that HIV-1^+^ microglia have been detected in cART-suppressed individuals (4), it is highly plausible that persistently infected microglia produce proinflammatory cytokines and chemokines, such as IFN-I and IP-10, contributing to a chronic state of neuroinflammation. Previous studies have suggested that IFN-I production contributes to cognitive impairments in HIV-1 infection (46) and neurodegenerative diseases (47, 48). Although multiple roles for chemokines in CNS inflammation have been described, CCL2, specifically, has been shown to modulate neuronal death in a mouse model (49, 50). Elevated levels of IP-10 have been observed in several neurodegenerative diseases, including in patients with HAND (51), and are known to affect neuronal viability (52, 53). Since we did not find obvious neuronal cytotoxicity in hiMG-hiNeuron cocultures in 6 days of infection, future studies will be focused on long-term cocultures and the consequence of persistent HIV-1 infection in microglia on neuronal cytotoxicity such as synaptic loss and dendrite degeneration (54).

### HIV icRNA and innate immune responses.

While viral proteins such as Tat, Vpr, and gp120 have been hypothesized to contribute to HIV-associated neuroinflammation (14), most of these studies relied on overexpression of viral proteins or transgenic animals. In this study, we showed that HIV-1-infection-induced activation of microglia in all primary cell culture models was triggered by cytoplasmic export of icRNA, since infection with HIV expressing a Rev mutant deficient for CRM1 interaction (M10) was unable to induce innate immune activation (Fig. 2), and CRM1 inhibitors suppressed HIV-induced activation in microglia (Fig. 2, 3, and 5). We previously showed that HIV icRNA expression alone induces IFN-I-dependent proinflammatory responses in MDMs, even though HIV icRNA expression does not lead to production of new virions or functional viral proteins, including gp120 and Vpr (8). Furthermore, the Rev mutant M10, which fails to induce innate immune activation in microglia, expresses multiply spliced viral RNAs, including those encoding Tat, suggesting that *de novo* Tat expression is not the trigger for HIV-induced microglia activation. Interestingly, HIV icRNA (*gag* mRNA) has been detected in the CSF from HIV-1^+^ individuals on cART (3, 10–12), and a highly sensitive RNAScope assay has revealed the presence of a significant number of SIV gag mRNA (icRNA)-positive cells in the brain of cART-suppressed monkeys (55). We postulate that these viral icRNA-expressing cells in the brain, which are most likely microglia, induce proinflammatory cytokines and affect neuronal health in cART-suppressed individuals. Several drug candidates that suppress expression or stability of HIV icRNA, such as Tat and Rev inhibitors (56, 57) or inhibitors that selectively target CRM1-dependent nuclear export of HIV icRNA (58), might have clinical benefit for suppressing HIV icRNA-induced aberrant inflammation and incidence of HAND in cART-suppressed patients.

### Establishment of primary human microglia culture system for HIV infection studies.

In order to investigate the role of HIV-1 infection of microglia in HIV-1 neuropathogenesis, and to overcome the limited access to primary microglia, we employed three different *in vitro* models of primary microglia in this study, MDMG, iCell-MG, and hiMG. MDMG expressed microglia-specific markers such as P2RY12 and were poorly susceptible to HIV-1 infection (Fig. 1). Since peripheral blood monocytes are readily accessible and the protocol for MDMG generation is relatively simple, MDMG is a reasonable model to study HIV-1 biology in microglia. It should be pointed out that infection of MDMG with HIV-1 in the absence of SAMHD1 antagonism was inefficient (Fig. 1). Further optimization of the generation protocol is warranted, for example, using M-CSF instead of GM-CSF in the differentiation conditions, since GM-CSF has been shown to induce antiviral SAMHD1 expression in MDMs (24) (Fig. 1G). To better mimic the origin of microglia (yolk-sac-derived), we used two independent iPSC-derived microglia lines and tested their susceptibility to replication-competent HIV-1 *in vitro*. iCell-MGs are commercially available and expressed microglia markers IBA-1 and P2RY12 (Fig. 3). It should be noted that in contrast to CNS-resident microglia (23, 41), we observed mostly intracellular expression of the microglia-specific marker P2RY12 in iCell-MGs (Fig. 3B). iCell-MGs were highly susceptible to HIV-1 infection (Fig. 3), which is in agreement with previous studies using primary fetal microglia (17). While iCell-MG is a powerful tool to study HIV-1 infection in microglia, the inability to genetically manipulate these cells limits their utility in robust mechanistic approaches.

The third model we used was hiMG-hiNeuron cocultures that were generated from iPSCs. This system has numerous advantages: (i) hiMGs are highly susceptible to HIV-1 infection (Fig. 5), (ii) establishment of iPSC-derived microglia and neuron cocultures allows for the study of intricate interactions between diverse cell types in the context of viral infection and, importantly, the impact of HIV-infection-induced microglia activation can be assessed on autologous neurons, (iii) the purinergic receptor, P2RY12, which detects extracellular nucleotides accompanied with CNS injury and regulates microglial homeostasis (41, 59, 60) and plays an important role in communicating with neighboring neurons to protect their functions (61), is robustly expressed on the hiMG cell surface (in contrast to the mostly intracellular expression of P2RY12 in iCell-MGs), (iv) iPSCs are amenable to gene-editing approaches (62), and (v) iPSC lines generated from somatic cells of various individuals, including HIV-infected patients, make possible studies of HIV infection of microglia from unique genetic backgrounds and their contribution to human disease. A recently published study (while the manuscript was in preparation) described a new cellular platform that consists of iPSC-derived microglia, neurons, and astrocyte tri-cultures (63) and showed that HIV-1 infection of iPSC-microglia in isolation or in tri-cultures resulted in production of proinflammatory cytokines, including IL-1β and tumor necrosis factor alpha (TNF-α). Though the mechanism of induction of proinflammatory responses in HIV-1-infected microglia was not defined, inflammatory responses were suppressed upon treatment with RT inhibitor (efavirenz) (63). Differentiation protocols for iPSC-derived microglia in this recently published study (63) were similar to those utilized for generation of iCell-MG (iCell microglia; Fujifilm Cellular Dynamics) that we tested for this report. While the cytokine-driven differentiation protocol generated iPSC-microglia with similar transcriptional profiles to human primary microglia (36, 63), our results suggest that iCell-MGs express low levels of P2RY12 on the cell surface, unlike primary human microglia (23, 41). Since the CNS environment is critical for establishing and maintaining microglial cell identity (64), coculture-dependent terminal differentiation of iPSC-microglia, as described here and by Takata et al. (39), may better model primary microglia in the brain.

### Impact of innate immune activation on homeostatic functions of microglia.

We have shown that HIV-1 infection of microglia promotes microglia cell death and proinflammatory cytokine production in the hiMG-hiNeuron cocultures (Fig. 5), though significant cytotoxicity of cocultured neurons was not observed at the time of harvest (6 days p.i.). In contrast, a recent study using nonisogenic iPSC-derived microglia and neurons (from independent lines) demonstrated that infected microglia induce neuronal death, and damaged neurons induce activation of HIV-1 transcription in latently infected microglia (65). These differences might be the result of a divergent experimental setup, as the hiMGs in this study were generated by coculturing hiMACs and hiNeurons from the same iPSC-line, and infections of hiMGs were initiated in cocultures. Further studies are needed to determine the effects of long-term coculture of HIV-infected hiMGs and hiNeurons and the consequences of persistent HIV icRNA-induced chronic inflammation on neuronal homeostasis. It has been shown that activation of microglia leads to dysfunctions such as defects in clearing neurotoxins, including fibrilar amyloid β and Tau, and promoting a senescent phenotype in microglia (reviewed in reference 14). Inclusion of other cell types which have been reported to be HIV-1^+^ in the CNS, such as astrocytes and perivascular macrophages (reviewed in reference 66), in the hiMG-hiNeuron coculture might better mimic the brain environment. In addition, human iPSC-derived cerebral organoids with diverse cell types that interact in a 3D environment are an attractive model to study HIV neuropathogenesis *in vitro* (67). Future studies will need to assess the effects of persistent HIV-1 infection on homeostatic functions of microglia and the contribution to neuronal dysfunction in these three-dimensional (3D) cerebral organoid cultures. Finally, our findings highlight the urgent need to develop novel therapeutic strategies targeting cytosolic HIV icRNA expression to reduce HIV-induced neuroinflammation and incidence of HAND.

## MATERIALS AND METHODS

### Viruses.

HIV-1 replication-competent molecular clones, Lai/YU-2env, single-round reporter virus constructs, LaiΔenvGFP (green fluorescent protein [GFP] in place of the *nef* orf), and Rev-deficient LaiΔenvGFP-M10, have been described previously (8, 68, 69). Replication-competent viruses were derived from HEK293T cells via calcium phosphate-mediated transient transfection (70). Single-round-replication-competent viruses pseudotyped with VSV-G were generated from HEK293T cells via cotransfection of HIV-1Δenv proviral plasmids and VSV-G expression plasmid and the packaging construct (psPAX2), if necessary (70). SIV_mac_ Vpx-containing VLPs were generated from HEK293T cells via cotransfection of SIV3^+^, an SIV packaging plasmid containing SIV_mac239_ Vpx (29), and VSV-G expression plasmid. Virus-containing cell supernatants were harvested 2 days posttransfection, cleared of cell debris by centrifugation (300 × *g*, 5 min), passed through 0.45-µm filters, and purified and concentrated by ultracentrifugation on a 20% sucrose cushion (24,000 rpm and 4°C for 2 hours with an SW32Ti or SW28 rotor [Beckman Coulter]). The virus pellets were resuspended in phosphate-buffered saline (PBS), aliquoted, and stored at −80°C until use. The capsid content of HIV-1 was determined by a p24^gag^ ELISA (70), and virus titer was measured on TZM-bl by measuring β-galactosidase (β-Gal) activity as previously described (71).

### Cell culture.

HEK293T (ATCC) and TZM-bl (NIH AIDS Reagent Program) were maintained in Dulbecco modified Eagle medium (DMEM) (Gibco) containing 10% heat-inactivated fetal bovine serum (FBS) (Gibco) and 1% pen/strep (Gibco) (37, 70, 72). THP-1 (NIH AIDS Reagent Program) was maintained in RPMI 1640 (Gibco) containing 10% FBS and 1% pen/strep (73). In some experiments, THP-1 cells were stimulated with phorbol myristate acetate (PMA) (Sigma-Aldrich) for 48 hours at 100 nM. All cell lines were tested for mycoplasma contamination and confirmed negative. Human iPSC-derived microglia were either purchased (iCell microglia; Fujifilm Cellular Dynamics) or generated by us (hiMG, see below). iCell microglia (iCell-MG) were maintained per the manufacturer’s instructions. All the reagents used to maintain iCell-MG are listed as follows: DMEM/F-12, HEPES no phenol red (Gibco; catalog no. 11039021), B-27 supplement (Gibco; catalog no. 17504044), GlutaMAX supplement (Gibco; catalog no. 35050061), insulin-transferrin-selenium (Gibco; catalog no. 41400045), minimal essential medium (MEM) nonessential amino acids (Gibco; catalog no. 11140050), penicillin-streptomycin (Gibco; catalog no. 15140122), N-2 supplement (Gibco; catalog no. 17502048), bovine serum albumin (Sigma-Aldrich; catalog no. A1470), recombinant human CD200 (ACRO Biosystems; catalog no. OX2-H5228), recombinant human IL-34 (PeproTech; catalog no. 200-34), recombinant human fractalkine (PeproTech; catalog no. 300-31), human insulin solution (Sigma-Aldrich; catalog no. I9278), human transforming growth factor-β1 (TGF-β1) (Miltenyi Biotec; catalog no. 130-095-066), ascorbic acid (Sigma-Aldrich; catalog no. A8960), recombinant human macrophage colony stimulating factor (M-SCF; PeproTech; catalog no. 300-25), and 1-thioglycerol (MTG) (Sigma-Aldrich; catalog no. M6145).

### Generation of monocyte-derived microglia-like cells and macrophages.

To generate monocyte-derived microglia (MDMG), CD14^+^ peripheral blood monocytes positively isolated with CD14 microbeads (Miltenyi Biotec) (68) were seeded on Geltrex-coated (Gibco) tissue culture plates and cultured for 12 to 14 days in RPMI 1640 Glutamax (Gibco) supplemented with 1% pen/strep, 100 µg/ml of IL-34 (PeproTech), and human GM-CSF (10 ng per ml; Miltenyi Biotec). Human monocyte-derived macrophages (MDMs) were derived from CD14^+^ peripheral blood monocytes by culturing in RPMI 1640 (Gibco) containing 10% heat-inactivated human AB serum (Sigma-Aldrich), and recombinant human M-CSF (20 ng per ml; PeproTech) for 5 to 6 days and maintained in the same medium.

### Generation of human iPSC-derived cells.

Human iPSCs were generated from human peripheral blood mononuclear cells (PBMCs) by using the STEMCCA polycistronic lentiviral vector (74, 75) followed by the removal of integrated reprogramming cassette using Cre recombinase (76) and were maintained in mTeSR1 medium (Stemcell Technologies). Human iPSC-derived primitive macrophages (hiMacs) were generated as previously reported (Fig. 4A) (39). Briefly, human iPSC colonies were specified to the mesoderm and induced into hemangioblast and toward hematopoietic precursors followed by differentiation into primitive macrophages by changing the culture medium every 2 to 4 days. After differentiation (day 26), floating cells were collected and used for fluorescence-activated cell sorting (FACS) as described below. In parallel, human iPSC-derived neurons (hiNeurons) were generated from the same batch of iPSCs as previously reported (39). Human iPSCs were dissociated to single cells, plated onto Matrigel-coated 6-well plates, and differentiated into neuronal progenitors (NPCs). NPCs were terminally differentiated into hiNeurons. To generate iPSC-derived microglia cells (hiMGs), CD45^+^ CD11b^+^ CD163^+^ CD14^+^ CX3CR1^+^ hiMacs were sorted by FACS as described below and cocultured with terminally differentiated hiNeurons for 14 days. All the reagents used to generate iPSC-derived cells are listed as follows: mTeSR (Stemcell Technologies; catalog no. 85850), ReLeSR (Stemcell Technologies; catalog no. 05872), DMEM/F-12, HEPES (Gibco; catalog no. 11330057), Iscove’s modified Dulbecco’s medium (IMDM; Gibco; catalog no. 12440061), Stempro-34 serum-free medium (SFM; Gibco; catalog no. 10639-011), neurobasal (Gibco; catalog no. 21103049), PBS (Gibco; catalog no. 14190-144), Ham’s F-12 nutrient mix (Gibco; catalog no. 11765054), N2 supplement (Gibco; catalog no. 17502048), B-27 supplement, serum free (Gibco; catalog no. 17504044), B27 minus vitamin A (Gibco; catalog no. 12587010), bovine albumin fraction V (7.5% solution) (Gibco; catalog no. 15260037), primocin (InvivoGen; catalog no. ant-pm-2), GlutaMax (Gibco; catalog no. 35050061), laminin (Gibco; catalog no. 23017-015), Matrigel hESC-qualified matrix (Corning; catalog no. 354277), Matrigel membrane matrix (Corning; catalog no. 354234), poly-l-ornithine solution (Sigma-Aldrich; catalog no. P4957), laminin mouse protein, natural (Gibco; catalog no. 23017015), human transferrin (Roche; catalog no. 10-652-202-001), glutamic acid (Sigma-Aldrich; catalog no. G1251), ascorbic acid (Sigma-Aldrich; catalog no. A4544), SB431542 (Tocris; catalog no. 1614), Y27632 (Rho-associated protein kinase [ROCK] inhibitor) (Stemgent; catalog no. 04-0012-02), MTG (Sigma-Aldrich; catalog no. M6145), Accutase (Gibco; catalog no. A1110501), polyornithine (Sigma-Aldrich; catalog no. P4957), CHIR99021 (Tocris; catalog no. 4423/10), γ-secretase inhibitor XXI, compound E (Millipore; catalog no. 565790), recombinant human brain-derived neurotrophic factor (BDNF; R&D Systems; catalog no. 248-BD), recombinant human glial cell line-derived neurotrophic factor (GDNF; R&D Systems; catalog no. 212-GD), recombinant human BMP-4 (R&D Systems; catalog no. 314-BP), recombinant human vascular endothelial growth factor (VEGF; R&D Systems; catalog no. 293-VE), recombinant human EGF (R&D Systems; catalog no. 236-EG), recombinant human fibroblast growth factor 2 (FGF2) (R&D Systems; catalog no. 233-FB), recombinant human stem cell factor (SCF; R&D Systems; catalog no. 255-SC), recombinant human DKK-1 (R&D Systems; catalog no. 5439-DK), recombinant human IL-3 (R&D Systems; catalog no. 203-IL), recombinant human IL-6 (R&D Systems; catalog no. 206-IL), and recombinant human M-CSF (R&D Systems; catalog no. 216-MC).

### Infection.

Cells were spinoculated with HIV-1 (1 h at room temperature [RT] and 1,100 × *g*) at various multiplicities of infection (MOI, typically 0.5 to 2), cultured for 2 to 3 h at 37°C, washed to remove unbound virus particles, and cultured for 3 to 6 days. Infection was quantified by analyzing p24^Gag^ released into the culture supernatants or GFP expression by flow cytometry (BD LSRII). In some experiments, cells were pretreated prior (at least 30 min) to infection with efavirenz (1 µM; NIH AIDS Reagent Program), raltegravir (30 µM; Selleck Chemicals), or treated 2 to 3 h postinfection (p.i.) with KPT-330 (1 µM, selinexor; Selleck Chemicals), or KPT-335 (0.1 µM, verdinexor; Selleck Chemicals). DMSO (Sigma-Aldrich) was used as a vehicle control.

### RNA analysis.

Total mRNA was isolated from 0.5 × 10^6^ to 1 × 10^6^ cells using an RNeasy kit (Qiagen) and reverse-transcribed using oligo(dT)_20_ primer (Superscript III; Invitrogen). Target mRNA was quantified using Maxima SYBR green (Thermo Scientific) using the following primer sets: P2RY12 (forward: 5′-CTTTCTCATGTCCAGGGTCAG-3′, reverse: 5′-CTGCAGAGTGGCATCTGGTA-3′) and GAS6 (forward: 5′-CCTTCCATGAGAAGGACCTCGT-3′, reverse: 5′-GAAGCACTGCATCCTCGTGTTC-3′). Primer sequences for GAPDH, HIV spliced RNA, and IP-10 were described previously (72). For hiMG-hiNeuron coculture, target mRNA was quantified using TaqMan universal PCR master mix (Thermo Fisher Scientific) and the following primer/probe sets: Hs99999905_m1 (GAPDH), Hs01922583_s1 (CX3CR1), Hs01881698_s1 (P2RY12), and Hs01881698_s1 (P2RY12). The threshold cycle (*C_T_*) value was normalized to that of GAPDH and represented as a relative value to a control using the 2^-ΔΔ^*^C^*_T_ method as described (72, 77). NanoSting analysis was performed using a human neuroinflammation kit and total RNAs (100 ng) isolated from MDMGs per the manufacturer’s instructions.

### ELISA.

IP-10 and CCL2 production in culture supernatants was measured with a BD human IP-10 ELISA set and a BD human MCP-1/CCL2 ELISA set, respectively. To quantitate virus production, p24^Gag^ in culture supernatants was quantified by in-house ELISA (8).

### Flow cytometry.

To sort CD45^+^ CD11b^+^ CD163^+^ CD14^+^ CX3CR1^+^ hiMacs, cells were stained with fixable viability stain 780 (BD Bioscience; catalog no. 565388) followed by staining with a phycoerythrin (PE)-conjugated mouse anti-human CD45 antibody (BD Biosciences; catalog no. 555483; 1:10), an allophycocyanin (APC)-conjugated anti-human CD11b antibody (BioLegend; catalog no. 301410; 1:20), a BV421-conjugated mouse anti-human CD14 antibody (BD Biosciences,; catalog no. 565283; 1:20), a fluorescein isothiocyanate (FITC)-conjugated mouse anti-human CD163 antibody (BD Biosciences; catalog no. 563697; 1:20), and a PerCP/Cy5.5-conjugated anti-human CX3CR1 antibody (BioLegend; catalog no. 341614; 1:20) in the presence of human Fc blocker (BD Bioscience; catalog no. 564220). Stained cells were sorted with Beckman Coulter MoFlo Astrios. To examine microglia activation, iCell-MGs or hiMG-hiNeuron cocultures were harvested with Cellstripper (Corning) and stained with Zombie-NIR (BioLegend; catalog no. 423105; 1:250) followed by staining with a BV421-conjugated mouse anti-P2RY12 antibody (BioLegend; 1:50) in the presence of human Fc blocker (BD Bioscience; catalog no. 564220). Cells were fixed with 4% paraformaldehyde (PFA) (Boston Bioproducts) for 30 min and permeabilized with Perm/Wash (BD Biosciences), and intracellular p24^Gag^ expression was detected as described (72) using an FITC-conjugated mouse anti-p24^Gag^ monoclonal antibody (KC57; Coulter; catalog no. 6604665; 1:25). As for iCell-MGs, cell surface CD169 expression was also analyzed using a BV605-conjugated mouse anti-CD169 antibody (BioLegend; 1:50). Intracellular tubulin β3 in the hiMG-hiNeuron cocultures was analyzed with an Alexa 549- or 647-conjugated mouse anti-tubulin β3 antibody (TUJ-1; BioLegend; 1:50). Cells were analyzed with BD LSRII (BD). Data were analyzed with FlowJo software (FlowJo).

### Imaging.

For MDMGs and iCell-MGs, cells cultured in coverslip chambers (LabTekII) were washed and fixed with 4% paraformaldehyde. Cells were then permeabilized with 0.1% TritonX100 and stained with a rabbit anti-P2RY12 antibody (Sigma-Aldrich; HPA014518; 1:100) or a rabbit anti-IBA1 antibody (Fujifilm Wako; catalog no. 019-19741; 1:250). Cells were then stained with Alexa594-conjugated anti-rabbit-IgG antibody (Invitrogen; catalog no. A-11072; 1:200) and DAPI (4′,6-diamidino-2-phenylindole; Sigma-Aldrich). Cells were analyzed with a Nikon SP5 confocal microscope. hiMG-hiNeuron coculture was fixed, permeabilized, and stained with a mouse anti-beta-tubulin III antibody (clone TUJ1; Stemcell Technologies; catalog no. 60052; 1:1000) and a rabbit polyclonal anti-TMEM119 antibody (Novus Biologicals; catalog no. NBP2-30551; 0.25-2 µg/ml) or a goat anti-IBA-1 antibody (Abcam; catalog no. ab5076; 1:500), followed by an Alexa Fluor 488-conjugated donkey anti-mouse IgG (Invitrogen; catalog no. A21202; 1:500) and an Alexa Fluor 594-conjugated goat anti-rabbit IgG (Invitrogen; catalog no. A11012; 1:500) or an Alexa Fluor 594-conjugated rabbit anti-goat IgG (Invitrogen; catalog no. A11080; 1:500), respectively, and DAPI (NucBlue; Invitrogen). Cells were analyzed with a Keyence BZ-X710 all-in-one fluorescence microscope. Images were analyzed with ImageJ (NIH).

### Immunoblot analysis.

To assess expression of host proteins, cell lysates containing 15 to 30 µg total protein were separated by SDS-PAGE and transferred to nitrocellulose membranes, and the membranes were probed with the following antibodies: a mouse anti-SAMHD1 antibody (Abcam; catalog no. ab67820; 1:1,000) or a rabbit anti-phosphorylated (Thr 592) SAMHD1 antibody (Cell Signaling; catalog no. 15038; 1:1,000) and specific staining visualized with secondary antibodies, goat anti-mouse-IgG-DyLight 680 (Pierce), or a goat anti-rabbit-IgG-DyLight 800 (Pierce). As loading controls, actin expression was probed using a rabbit anti-actin antibody (Sigma-Aldrich; A2066; 1:5,000). Membranes were scanned with an Odessy scanner (Li-Cor).

### Statistics.

All the statistical analysis was performed using GraphPad Prism 8. *P* values were calculated using one-way analysis of variance (ANOVA) followed by the Tukey-Kramer posttest (symbols for *P* values shown with a line) or Dunnett’s posttest (comparing to mock, symbols for *P* values shown on each column), One sample *t* test (comparing two samples, symbols for two-tailed *P* values shown with a line) or a Wilcoxon signed rank test (comparing two samples, symbols for two-tailed *P* values shown with a line). *, *P* < 0.05; **, *P* < 0.01; ***, *P* < 0.001; ****, *P* < 0.0001. No symbol: not significant (*P* ≥ 0.05).

### Data availability.

We declare that the data that support the findings of this study are available within the paper and from the corresponding author upon reasonable request.
